# Explaining Accurate Predictions of Multitarget Compounds with Machine Learning Models Derived for Individual Targets

**DOI:** 10.3390/molecules28020825

**Published:** 2023-01-13

**Authors:** Alec Lamens, Jürgen Bajorath

**Affiliations:** Department of Life Science Informatics, B-IT, LIMES Program Unit Chemical Biology and Medicinal Chemistry, Rheinische Friedrich-Wilhelms-Universität, Friedrich-Hirzebruch-Allee 5/6, D-53115 Bonn, Germany

**Keywords:** multitarget compounds, single-target compounds, machine learning, activity prediction, model explanation, feature analysis

## Abstract

In drug discovery, compounds with well-defined activity against multiple targets (multitarget compounds, MT-CPDs) provide the basis for polypharmacology and are thus of high interest. Typically, MT-CPDs for polypharmacology have been discovered serendipitously. Therefore, over the past decade, computational approaches have also been adapted for the design of MT-CPDs or their identification via computational screening. Such approaches continue to be under development and are far from being routine. Recently, different machine learning (ML) models have been derived to distinguish between MT-CPDs and corresponding compounds with activity against the individual targets (single-target compounds, ST-CPDs). When evaluating alternative models for predicting MT-CPDs, we discovered that MT-CPDs could also be accurately predicted with models derived for corresponding ST-CPDs; this was an unexpected finding that we further investigated using explainable ML. The analysis revealed that accurate predictions of ST-CPDs were determined by subsets of structural features of MT-CPDs required for their prediction. These findings provided a chemically intuitive rationale for the successful prediction of MT-CPDs using different ML models and uncovered general-feature subset relationships between MT- and ST-CPDs with activities against different targets.

## 1. Introduction

In drug discovery, compounds with multitarget (MT) activity (also termed promiscuous compounds) are required for the polypharmacology-driven treatment of multifactorial diseases [1,2,3,4]. Polypharmacology refers to the MT engagement of small molecules and the resulting in vivo effects [4,5,6]. MT compounds (MT-CPDs) form well-defined (pseudo-specific) interactions with different targets. While polypharmacological compounds were often serendipitously discovered, computational approaches are increasingly used to support the identification or design of desirable MT-CPDs [6,7,8,9]. For example, machine learning (ML) models were derived on the basis of chemical structures to systematically distinguish between MT-CPDs and corresponding single-target compounds (ST-CPDs) [10,11]. Furthermore, random forest (RF) predictions on a molecular test system, comprising MT- and ST-CPDs for 170 target pairs, have shown that structural features distinguishing MT- and ST-CPDs depend on target pairs and have no potential for generalization [12]. This complicates MT-CPD design because generally applicable design strategies will be difficult, if not impossible to devise. On the other hand, the ability to develop ML models that are capable of detecting MT-CPDs enables computational screening campaigns to prioritize candidate compounds for polypharmacology. Although proof-of principle has been established for predicting MT-CPDs, ML models are still subject to further refinement, especially for specific target combinations of interest. This will also require a better understanding of their learning characteristics. To these ends, explainable ML (XML) methods can be considered. The application of XML approaches makes it possible to understand how compound activity predictions are determined for ML models of any complexity. This typically involves the identification of (molecular representation) features making the largest contributions to correct predictions of, for example, active vs. inactive compounds or—as in our current study—MT- vs. ST-CPDs. For different classification models, features that are decisive for class label predictions can be compared to rationalize model decisions and differences between them.

Herein, we have derived and compared different ML models for predictions of MT- and the corresponding ST-CPDs, using a new compound test system covering functionally related and distinct targets. These calculations yielded some surprising results. To rationalize these predictions and test hypotheses derived from them, an XML approach was applied, providing detailed and chemically intuitive explanations for model decisions on MT- and the corresponding ST-CPDs.

## 2. Results and Discussion

### 2.1. Compound Test System and Random Forest Models

To set up a test system for MT- and ST-CPD predictions, we carried out a systematic search for functionally distinct target pairs (i.e., targets with unrelated biological functions; see Section 3), for which sufficient numbers of MT- and corresponding ST-CPDs were available for model building (see Section 3). In our current analysis, MT-CPDs refer to compounds with confirmed activity against two targets. Pairs of distinct targets with available MT-CPDs are generally rare. We were able to identify 10 qualifying pairs of functionally distinct targets and complemented this set with 10 pairs of functionally related targets. In this case, many qualifying pairs were available. To guide the search, we built initial RF models for each pair to distinguish between MT- and ST-CPDs, and we selected the target pairs with the highest prediction accuracy. The 20 target pairs comprising the test system are reported in Table 1. The choice of RF and the prediction accuracy-oriented selection of functionally related target pairs was motivated to provide a basis for XML analysis, for which RF is particularly suitable; this is because an accurate XML algorithm is available for this method (see Section 3). Furthermore, since XML generally benefits accurate predictions and robust (stable) predictions, the simplest ML method that yields high prediction accuracy should typically be selected for a given prediction task.

For each target pair, two final RF models were developed, including an (1) ST model to distinguish ST-CPDs from randomly chosen compounds with other activity (assumed to be inactive against the targets of a pair), and (2) MT model to distinguish MT- and ST-CPDs. In addition, as a control, each ST model was also used to predict MT-CPDs for the target pair. Accordingly, the primary prediction tasks included ST-CPDs vs. inactive compounds and ST- vs. MT-CPDs, for which different models were derived. The model, trained to distinguish between ST-CPDs and inactive compounds, was then also used to predict MT-CPDs vs. inactive compounds. Features determining the predictions of each model were then identified using the XML methodology, as detailed below.

### 2.2. ST and MT Model Performance

**Figure 1:**
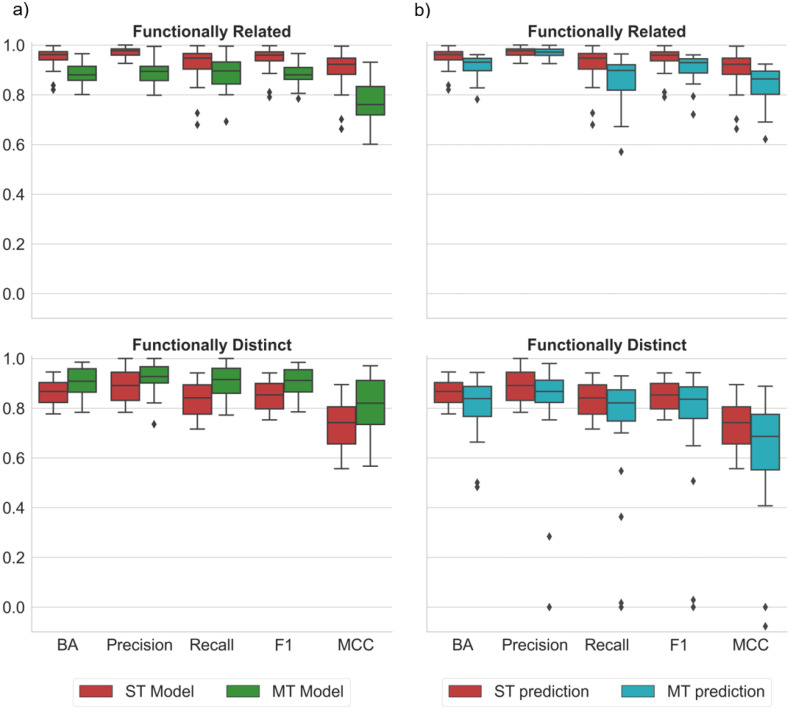
Performance of RF models for ST- and MT-CPD prediction. Boxplots report the distributions of different performance measures for predictions on all target pairs averaged over 10 independent trials. Performance measures included balanced accuracy (BA), precision, recall, F1, and Matthews correlation coefficient (MCC). Compounds with activity against pairs of functionally related or distinct targets were predicted. (**a**) ST model (red), trained for classification of randomly chosen vs. ST-CPDs; MT model (green), trained for classification of ST- vs. MT-CPDs. (**b**) ST model, applied to classify randomly chosen vs. ST-CPDs (same as in (**a**), red) and randomly chosen vs. MT-CPDs (cyan). In boxplots, diamond symbols represent statistical outliers.

Figure 1 summarizes the results of our systematic predictions. Both the ST and MT models achieved a high prediction accuracy on the basis of different performance measures. Notably, a high prediction accuracy was not only observed for the pre-screened pairs of related targets, but also for the pairs of distinct targets. The accuracy was only slightly higher for related than distinct targets and was overall comparable for the ST and MT models (Figure 1a). A balanced accuracy (BA), greater than 80 or 90%, high precision, and Matthews correlation coefficient (MCC), with values between 0.7 and 0.9, were consistently observed. The functionally related target pairs included closely related enzyme isoforms (Table 1), for which it was not necessarily expected that MT- and corresponding ST-CPDs were distinguished with high accuracy. Moreover, when the ST model from each pair was used to predict its MT-CPDs, comparably high precision accuracy was achieved, for both related and distinct targets. This finding was unexpected because the ST models were trained to distinguish ST-CPDs from randomly selected compounds, without taking any MT-CPDs into account.

As a plausible hypothesis, the ability of an ST model to accurately differentiate both ST- and MT-CPDs from randomly selected compounds might indicate that many ST-CPDs would be under-tested and also have MT activity. However, this conjecture was not consistent with the ability of MT models to accurately distinguish between MT- and corresponding ST-CPDs. Hence, further analysis of the ML results was required to better understand the ability of ST models to distinguish between MT-CPDs and randomly selected actives, and MT models to differentiate MT- from ST-CPDs.

### 2.3. Rationalizing Model Decisions

To better understand these findings, we applied the Shapley Additive exPlanations (SHAP) approach (see Section 3), a model-agnostic XML method that we adapted for explaining compound activity predictions (see Section 3). SHAP analysis quantifies the contribution of representation features that are present or absent in a test instance to individual predictions. Features that support or oppose a given prediction make positive and negative contributions (quantified by SHAP values), respectively, and the sum of all SHAP values gives the probability of the prediction. By summing feature contributions over individual test instances and their predictions, feature importance can be quantified across entire test sets, which is termed the cumulative SHAP analysis. In our predictions, chemical structure was represented using layered atom environments (see Section 3), which are topological structural descriptors.

**Figure 2:**
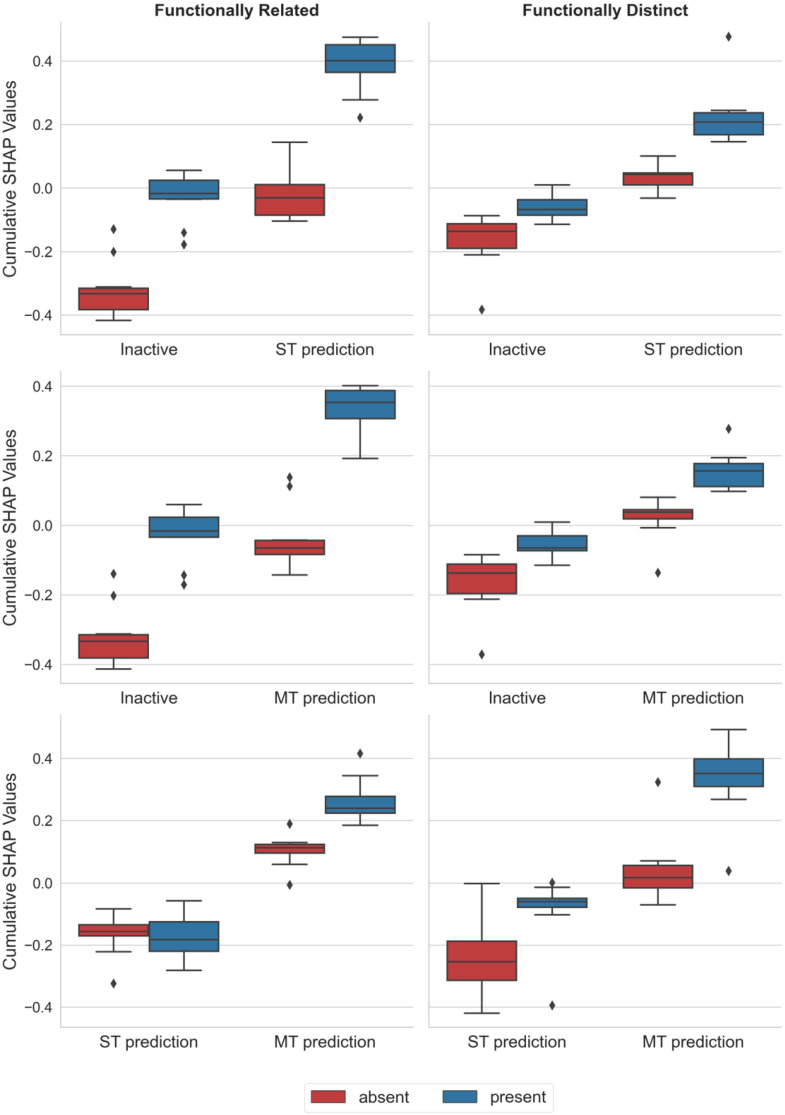
Cumulative SHAP analysis. Contributions of features absent (red) or present (blue) in test compounds were quantified using SHAP values across all correctly predicted randomly collected compounds (designated inactive), ST-, and MT-CPDs. Feature contributions are reported for three prediction tasks: ST model prediction of randomly selected vs. ST-CPDs (top), ST model prediction of random vs. MT-CPDs (middle), and MT model prediction of ST- vs. MT-CPDs (bottom).

Figure 2 shows the results of cumulative SHAP analysis for predictions of ST- and MT-CPDs using the ST model, and differentiation between ST- and MT-CPDs via the MT model.

For the prediction of ST- and MT-CPDs with the ST model, very similar feature contribution patterns were detected. For functionally related target pairs (on the left in Figure 2), ST-CPDs were distinguished from random (inactive) compounds based on features that were present in ST-CPDs, making strong positive contributions to prediction probability; however, these were absent in random compounds (making negative contributions). These features largely determined the correct prediction of ST-CPDs. By contrast, features present in random, and absent in ST-CPDs, made very little, if any contributions (with cumulative SHAP values close to zero). Essentially, the same observations were made when MT-CPDs were correctly predicted with ST models. Notably, the magnitude of positive and negative feature contributions was very similar in both cases. A somewhat different picture emerged for predictions with MT models. In this case, correct discrimination between MT- and corresponding ST-CPDs was also mostly driven by features that were present in MT- and absent in ST-CPDs. However, features absent in MT-CPDs also made smaller positive contributions, while features present or absent in ST-CPDs made negative contributions. Hence, prediction outcomes for MT models of related target pairs were determined by more complex feature patterns than for the corresponding ST models (while prediction accuracy was comparably high). For functionally distinct target pairs (on the right in Figure 2), the feature patterns of MT models changed again. Here, features present in MT-, but absent in the corresponding ST-CPDs, dominated correct predictions, very similar to the observations made for predictions with ST models for related target pairs. Again, the same trends were also observed for ST models of distinct target pairs, but the differences between contributions of features that were present or absent in ST- or MT-CPDs and random compounds were smaller.

Taken together, on the basis of the results in Figure 2, the cumulative SHAP analysis provided meaningful explanations for correct predictions of all ST and MT models. The analysis revealed a pivotal role for features present in ST-CPDs and MT-CPDs when they were distinguished from random compounds using ST models, as well as for features present in MT-CPDs when they were differentiated from the corresponding ST-CPDs with MT models. While differences between feature contributions varied in magnitude, depending on the target pairs, the interplay between feature presence and absence was a constant factor in determining correct predictions.

On the basis of these findings, a key question was how feature sets determining the prediction of ST- and MT-CPDs might be related to each other; this was addressed in the next step.

### 2.4. Comparative Feature Analysis

**Figure 3:**
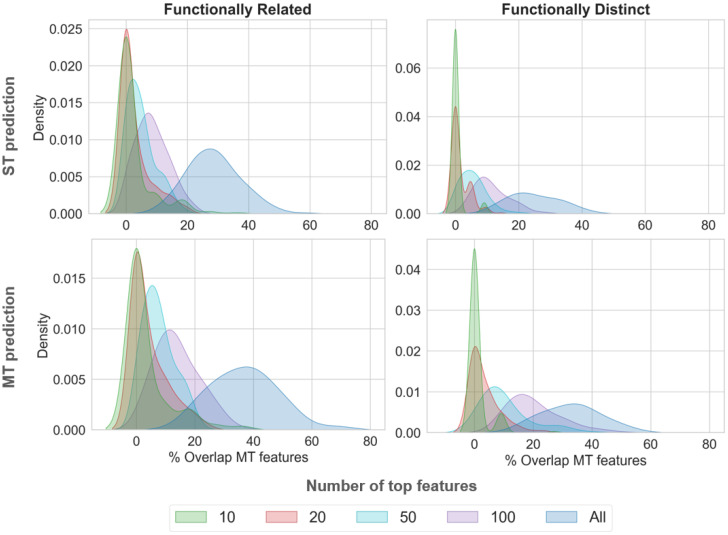
Feature overlap. Density plots show the overlap of feature sets of MT-CPDs, correctly predicted by the MT model, with feature sets of ST-CPDs, correctly predicted by the ST model (top) and MT-CPDs, correctly predicted by the ST model (bottom) for increasing numbers of top-ranked features. Feature overlap was calculated over all independent trials for related or distinct target pairs.

Sets of top-ranked features of increasing size that make the most important contributions to correct predictions with ST and MT models were compared, and their overlap was determined. Figure 3 shows the results. Feature overlap was already detected for small sets of top-ranked features and generally increased with the increasing size of the feature sets, as one would expect. Correct predictions of MT-CPDs with the MT model and ST model resulted in a larger overlap than predictions of MT-CPDs with the MT model, and ST-CPDs with the ST model. As expected, predictions for distinct target pairs yielded a smaller feature set overlap than for related target pairs, reflecting a typically lower similarity of compounds that are active against functionally distinct, rather than related, targets. The findings in Figure 3 clearly indicated that (i) feature sets determining predictions of MT- and ST-CPDs were overlapping yet distinct and (ii) MT- and ST-CPDs shared a subset of features determining the accurate prediction of MT-CPDs. In addition, we found that feature sets determining the prediction of ST- and MT-CPDs, using ST models, also had significant overlap, consistently between 40% and 60% for different numbers of top-ranked features.

Taken together, these results provided an intuitive explanation for the ability of ST models to differentiate between ST- or MT-CPDs and random compounds. MT- and corresponding ST-CPDs shared structural features that distinguished them from random compounds and were detected by ML. Furthermore, the results also explained why MT-CPDs were successfully distinguished from corresponding ST-CPDs. This was the case because MT- and corresponding ST-CPDs shared a subset of the features common to MT-CPDs. The additional features shared by MT-CPDs, then, determined their correct prediction using MT models.

### 2.5. Feature Mapping

**Figure 4:**
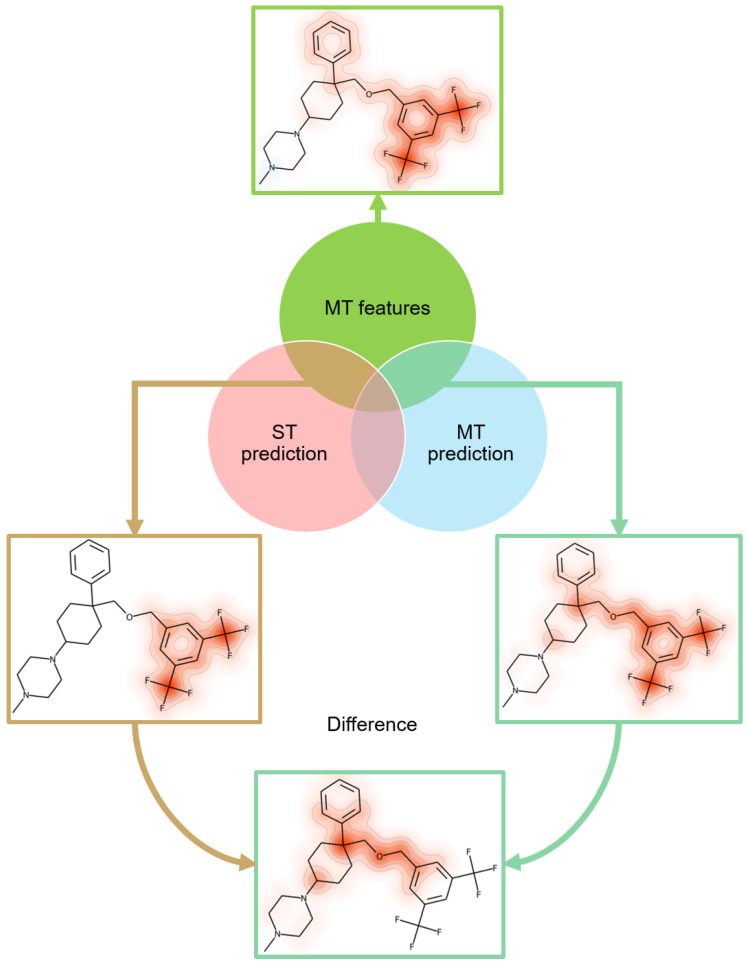
Feature mapping. For an exemplary MT-CPD that was correctly predicted using ST and MT models, different sets of features were mapped onto the structure. Green features at the top are from the MT models and the others represent overlapping features from the prediction of ST- and MT-CPDs using the ST model. Additional features from the MT-CPD prediction form the difference set at the bottom.

Subset relationships between features of MT- and ST-CPDs determining their correct predictions were further explored by mapping features on test compounds. As an example, in Figure 4, features positively contributing to the correct prediction of MT-CPDs for target pair (substance-P receptor/sodium-dependent serotonin transporter), using the MT model (green), were mapped onto a correctly predicted test compound (top). In addition, the subset of features overlapping with those determining the prediction of ST-CPDs (pink) or MT-CPDs (light blue), using the ST model, were mapped on the MT-CPD (middle left and right, respectively). Feature mapping revealed that the correct predictions of both MT- and ST-CPDs, using different models, were strongly supported by the di-substituted phenyl ring on the right of the compound, with feature centers on the two tri-fluoro methyl substituents. This was clearly indicated by the feature overlap for the ST model predictions. However, to distinguish between MT- and corresponding ST-compounds, additional features were required; these mostly encompassed the ether linker fragment adjacent to the ring. These features represented the MT-CPD-specific difference mapped at the bottom of Figure 4.

**Figure 5:**
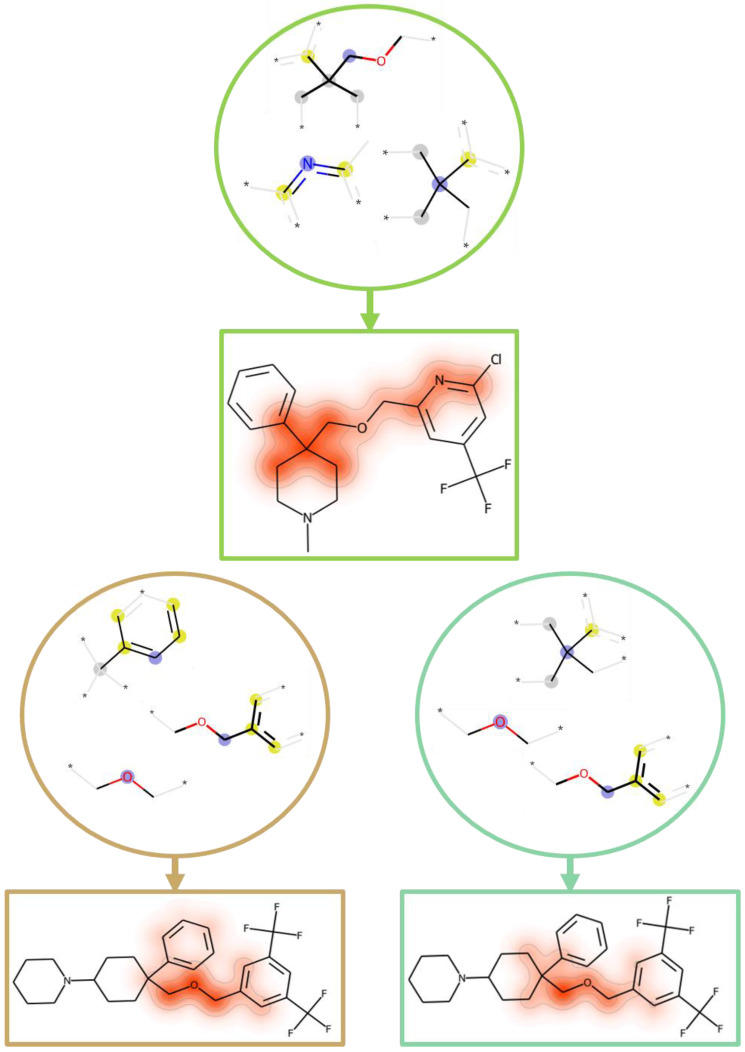
Most important features. At the top, the three most important features for correct predictions with the MT model are shown for the same set of MT-CPDs as in Figure 4. The features were mapped onto a compound with the largest cumulative SHAP values for them. Shown below are the top three features for the prediction of ST-CPDs (lower left) or MT-CPDs (lower right) using the ST model. These features were mapped onto another MT-CPD that contained them. The color code and representation are according to Figure 4. The different predictions shared one or two individual top-ranked features.

From the entire set of MT-CPDs for the target pair (substance-P receptor/sodium-dependent serotonin transporter), we extracted the three structural features that were most important for correct predictions using the MT model. In Figure 5 (top), these structural fragments were mapped onto an MT-CPD that had the largest cumulative SHAP values for these features. The three top-ranked features were also determined for predictions of ST- or MT-CPDs, using the ST model, and mapped onto another MT-CPD containing them (Figure 5, bottom left and right, respectively). Top-ranked features for the three predictions also overlapped, as illustrated in Figure 5. Additional features with positive contributions were required to determine correct predictions, forming sets with subset relationships, as discussed above.

### 2.6. Conclusions

In this work, we investigated different ML models for their ability to distinguish between MT- and ST-CPDs, or differentiate them from randomly selected active compounds. The ability of a model trained on ST-CPDs to correctly predict MT-CPDs was unexpected, especially because MT- and ST-CPDs were also distinguished with high accuracy. These findings were further investigated using an XML approach to rationalize the predictions. The analysis revealed that overlapping, yet distinct feature sets, were responsible for differentiating ST- and MT-CPDs from random compounds and provided a chemically intuitive explanation for the different prediction outcomes. Structural features, required to correctly predict ST-CPDs, represented the subsets of those required to predict MT-CPDs. Their prediction, using MT models, was then determined by additional features shared by MT-CPDs. Thus, MT-CPDs contained structural features of corresponding ST-CPDs and additional characteristics that set them apart from ST-CPDs, consistent with their MT activity. Thus, the XML analysis provided a general rationale for structural differences between MT- and the corresponding ST-CPDs of different target pairs on the basis of feature subset relationships. The ability of a ST models to correctly predict corresponding MT-CPDs, and the rationalization of this ability, also has broader implications for ML. For example, specifically derived ST models might also be tested on compounds with activity against closely related targets (from the same family), or on others, where the overlap of features determining predictions might be expected. In addition, the compounds prioritized in prospective applications, using ST models, might also be further investigated for MT activity, including related, as well as distinct, targets.

## 3. Materials and Methods

### 3.1. Compound Activity Classes and Target Pairs

Active compounds were extracted from CHEMBL (version 30) [13], based on different selection criteria. Compounds were required to have standard potency measurements (K_i_, K_d_ or IC_50_) and a numerically specified potency value (standard relation: “=”). In addition, only direct target interactions (target confidence score: 9) were considered. Potential assay interference compounds were removed using publicly accessible filters [14,15,16]. Compounds with interactions flagged as “not active”, “inactive”, “potential transcription error”, “inconclusive”, and compounds with potency values lower than 10 µm or a molecular mass of at least 1000 Da, were disregarded. The resulting compound dataset was analyzed to identify target pairs containing MT-CPDs, with reported activity against both targets of a pair, and the corresponding ST-CPDs, with reported activity against only one of these targets. To enable the derivation of ML models, target pairs were selected, for which more than 25 + 25 ST-CPDs (that is, 25 for each target) and 50 MT-CPDs were available. The resulting target pair datasets were supplemented with an equal number of randomly selected ChEMBL compounds. A total of 573 target pairs were obtained, representing 229,446 unique compounds. The 573 target pairs contained 10 pairs formed by functionally distinct targets (that is, targets with unrelated biological functions), following the standard UniProt target classification scheme [17]. For the remaining 563 pairs formed by functionally related targets, RF models were derived to distinguish ST- and MT-CPDs from random compounds (see below), and the top 10 pairs, for which highest prediction accuracy was achieved, were selected. Thus, a test system comprising 10 + 10 pairs of functionally related and distinct targets, respectively, was assembled (Table 1). For each pair, equal numbers of ST- and MT-CPDs were obtained by random sampling of the majority class, yielding a total of 18,450 unique compounds.

### 3.2. Molecular Representation

As a molecular representation, the unfolded version of the Morgan fingerprint, corresponding to the extended connectivity fingerprint [18], was generated using RDKit [15]. Layered atom environments, up to a bond radius of 2 (corresponding to a bond diameter of 4), were generated for each atom in a compound. These molecule-specific layered atom environments were used to generate hash values. Each unique hash value was assigned to a single position in a feature vector, thereby avoiding potential bit collisions and creating consistent bit-to-feature assignments for feature importance analysis.

### 3.3. Machine Learning

Classification models were developed using the RF implementation of scikit-learn [19]. RF is a supervised ML method that utilizes ensembles of decision trees, derived from bootstrapped training data, to obtain majority votes for class label prediction.

#### 3.3.1. Model Training, Hyperparameter Optimization, and Predictions

For each target pair, MT and ST models were derived and tested on 10 randomly selected partitions of 70% training and 30% test data. Model hyperparameters were optimized using corresponding training data splits. Hyperparameters included the number of decision trees (25, 50, 100, 200, 400), the minimal number of leaves required for a split (2, 3, 5, 10), and the minimal number of samples for a leaf node (1, 2, 5, 10). For other hyperparameters, default settings were used. The final model was built using optimized hyperparameters and the entire training set.

ST and MT models were applied to predict their original test sets. In addition, ST models were also applied to predict another test set comprising random compounds and MT-CPD’s.

#### 3.3.2. Performance Measures

The performance of the ST and MT models was assessed based on five measures, including balanced accuracy (*BA*) [20], precision, recall, *F*1 score (*F*1) [21] and Matthew’s Correlation Coefficient (MCC) [22].

$$BA=\frac{1}{2}\left(TPR+TNR\right)$$

$$\mathrm{precision}=\frac{TP}{TP+FP}$$

$$\mathrm{recall}=\frac{TP}{TP+FN}$$

$$F1=2\times \frac{TP}{2TP+FP+FN}$$

$$\mathrm{MCC}=\frac{TP\times TN-FP\times FN}{\sqrt{\left(TP+FP\right)\left(TP+FN\right)\left(TN+FP\right)\left(TN+FN\right)}}$$

### 3.4. Model Explanation

To rationalize predictions, the ML adaptation of the Shapley value concept from game theory [23] was applied. In ML, Shapley values quantify the contributions of individual features that are present or absent in a test instance for its prediction. Therefore, a local interpretation model is derived that approximates the ML model in a given region of feature space for individual predictions. The local approximation is termed the Shapley Additive exPlanations (SHAP) [24], which has been adapted to rationalize compound activity predictions [25]. The sum of all feature contributions, quantified by SHAP values, yields the probability of a prediction. For RF and other decision tree methods, the TreeExplainer algorithm has been introduced for the calculation of the exact SHAP values [26]. Thus, RF is particularly suitable for XML analysis. TreeExplainer was used here with “interventional” feature perturbation [26]. For each model, the entire training data served as the background sample.

Feature overlap between models for different prediction tasks was determined using all the correctly predicted test compounds. Here, only features that were present (bit status = on) in test compounds and positively contributed to their correct prediction were considered. To account for larger or smaller differences between the prediction probability and expected value [24], the resulting SHAP values were normalized by dividing them by the sum of the positive SHAP values for each compound. The overall weighted contribution of normalized SHAP values to the class label assignment was then calculated by taking the sum of each specific feature across all correctly predicted compounds and dividing it by the occurrence of the feature. This method yielded a vector with the weighted contribution values of each feature. To determine feature overlap, the intersection of the vectors for different prediction tasks was calculated. The intersection was then divided by the total amount of weighted positive features contributing to the prediction of a given class label.

## Figures and Tables

**Table 1:** Target pairs.

Functionally Related	Functionally Distinct
Sodium/glucose cotransporter 1 Sodium/glucose cotransporter 2	Tumor necrosis factor Nucleotide-binding oligomerization domain-containing protein 1
Carbonic anhydrase 4 Carbonic anhydrase 7	5-hydroxytryptamine receptor Sodium-dependent noradrenaline transporter
Carbonic anhydrase 4 Carbonic anhydrase 9	D2 dopamine receptor Sodium-dependent serotonin transporter
Cathepsin B Cathepsin S	Acetylcholinesterase Amine oxidase B
Insulin-like growth factor 1 receptor kinase ALK receptor tyrosine kinase	Acetylcholinesterase Beta-secretase 1
Histone deacetylase 2 Histone deacetylase 6	Substance-P receptor Sodium-dependent serotonin transporter
Histone deacetylase 3 Histone deacetylase 8	Amine oxidase B Adenosine receptor A2a
Coagulation factor X Plasminogen	D3 dopamine receptor Sodium-dependent serotonin transporter
Prothrombin Coagulation factor VII	Histamine H3 receptor Sodium-dependent serotonin transporter
Intestinal collagenase Collagenase 3	Histamine H1 receptor Sodium-dependent serotonin transporter

## Data Availability

Target pair-based compound datasets are available from the authors.

## Sample Availability

Target pair-based compound datasets are available from the authors.
